# Acetarsol Suppositories: Effective Treatment for Refractory Proctitis in a Cohort of Patients with Inflammatory Bowel Disease

**DOI:** 10.1007/s10620-017-4890-6

**Published:** 2018-02-19

**Authors:** Christopher J. Kiely, Angela Clark, Joya Bhattacharyya, Gordon W. Moran, James C. Lee, Miles Parkes

**Affiliations:** 1 Department of Gastroenterology, Addenbrooke’s Hospital, Cambridge University Hospitals, Cambridge, UK; 2 Pharmacy Department, Addenbrooke’s Hospital, Cambridge University Hospitals, Cambridge, UK; 3 Department of Medicine, University of Cambridge, Cambridge, UK; 4 National Institute for Health Research (NIHR) Biomedical Research Centre in Gastrointestinal and Liver Diseases, Nottingham University Hospitals NHS Trust and the University of Nottingham, Queens Medical Centre Campus, E Floor West Block, Derby Road, Nottingham, NG7 2UH UK

**Keywords:** Inflammatory bowel disease, Proctitis, Acetarsol, Crohn’s disease, Ulcerative colitis

## Abstract

**Background:**

Management of proctitis refractory to conventional therapies presents a common clinical problem. The use of acetarsol suppositories, which are derived from organic arsenic, was first described in 1965. Data concerning clinical efficacy and tolerability are very limited.

**Aim:**

To examine the efficacy of acetarsol suppositories for the treatment of refractory proctitis.

**Methods:**

A retrospective analysis was performed on patients with inflammatory bowel disease treated with acetarsol suppositories between 2008 and 2014 at Addenbrooke’s Hospital, Cambridge, United Kingdom. Clinical response was defined as resolution of symptoms back to baseline at the time of next clinic review.

**Results:**

Thirty-nine patients were prescribed acetarsol suppositories between March 2008 and July 2014 (29 patients with ulcerative colitis, nine with Crohn’s disease, and one with indeterminate colitis). Thirty-eight were included for analysis. The standard dose of acetarsol was 250 mg twice daily per rectum for 4 weeks. Clinical response was observed in 26 patients (68%). Of the 11 patients who had endoscopic assessment before and after treatment, nine (82%) showed endoscopic improvement and five (45%) were in complete remission (Wilcoxon signed-rank test *p* = 0.006). One patient developed a macular skin rash 1 week after commencing acetarsol, which resolved within 4 weeks of drug cessation.

**Conclusion:**

Acetarsol was effective for two out of every three patients with refractory proctitis. This cohort had failed a broad range of topical and systemic treatments, including anti-TNFα therapy. Clinical efficacy was reflected in significant endoscopic improvement. Adverse effects of acetarsol were rare.

## Introduction

Acetarsol suppositories are derived from organic arsenic, meaning that the arsenic atom is linked to carbon. Arsenic was first described as a treatment for proctitis in 1965 [1]. The mechanism of action is unknown. Data concerning clinical efficacy and tolerability of acetarsol suppositories are limited to two small studies (Connell et al. [1], 44 patients; Forbes et al. [2], ten patients). Acetarsol does have an antimicrobial effect and is been used for decades in the treatment of refractory bacterial vaginosis [3]. In refractory proctitis, the usual dose of acetarsol suppositories is 250 mg BD for 4 weeks, with at least a 4-week washout period prior to a second course, if this is required. Acetarsol is not currently approved by the FDA for use in the USA.

Here, we report our experience of using acetarsol suppositories in the management of 39 patients with refractory proctitis. Almost half of patients with ulcerative colitis have inflammation confined to the rectum [4], and in those with more extensive disease, rectal inflammation may contribute disproportionately to the burden of symptoms. Trials using conventional 5-aminosalicylic acid agents (5-ASAs) to treat proctitis have demonstrated these to be effective in 78–87% of patients [5, 6]; however, a significant minority do not respond—and indeed a hard core of such patients also appear resistant to both thiopurine therapy and biologic therapy [7, 8].

## Methods

A retrospective analysis was performed on patients with IBD who were prescribed acetarsol suppositories between 2008 and 2014 at Addenbrooke’s Hospital, Cambridge, United Kingdom. Patients were identified through the hospital pharmacy database. All patients who received acetarsol suppositories during this period were included. This included some patients who had more extensive disease than that confined to the rectum, but where the rectum was the maximally inflamed segment. When available, endoscopic data were collected. This was performed at the discretion of the clinician and consisted of either flexible sigmoidoscopy or rigid sigmoidoscopy. Mayo endoscopic sub-scores were determined retrospectively; these were based on endoscopic photos and written descriptions provided in the medical record. Clinical response was determined as an improvement in symptoms or reduction in the Mayo endoscopic sub-score by one or more point. Clinical remission was defined as symptoms (rectal bleeding, tenesmus, and urgency returning) to “normal” and/or an endoscopic sub-score of one or zero. Serum arsenic levels were not routinely measured. This study was conducted with the approval of the Human Research and Ethics Committee of Cambridge University Hospitals NHS Foundation Trust.

### Statistics

Statistical analysis was performed using SPSS version 24 (IBM) and R [9]. *P* values less than 0.05 were considered significant. Correction for multiple comparison was made using the Benjamini and Hochberg method using the stats package in R.

## Results

Thirty-nine patients received acetarsol suppositories during the study period (29 UC, nine Crohn’s disease, one indeterminate colitis). One patient who was treated for pouchitis was excluded from the analysis.

### Patient Characteristics

Demographic and prior treatment details are shown in Table 1. The first course of acetarsol was given, on average, 7 years following the initial diagnosis of IBD. Only patients with refractory disease were prescribed acetarsol; on average, they had failed medications from three different classes [mean 3.0, ± standard deviation (SD) 0.93]. The median duration for follow-up was 6.5 years ± SD 3.4 following the first use of acetarsol. Four patients were receiving anti-TNFα therapy at the time of the course of acetarsol, 21 (55%) were receiving thiopurines, seven (18%) were on methotrexate, and seven patients (18%) were prescribed concurrent corticosteroids (high-dose oral corticosteroids in four patients, while three were maintained on long-term low-dose corticosteroid regimens).

**Table 1:** Baseline characteristics of the patient cohort

	Total
Number of patients	38
Median age [years (± standard deviation)]	39.3 (± 15)
Sex	
Male	16 (42%)
Female	22 (58%)
Ulcerative colitis	29 (76%)
Crohn’s disease	9 (24%)
Median duration of disease (years)	6.9 (± 5.0)
Median follow-up (years)	6.4 (± 3.4)
Prior treatment	
5-ASA (oral)	29 (76%)
5-ASA (topical)	38 (100%)
Corticosteroids (systemic)	36 (95%)
Corticosteroids (topical)	34 (90%)
Thiopurines	31 (82%)
Methotrexate	13 (34%)
Anti-TNF agents	9 (24%)

**Fig. 1:**
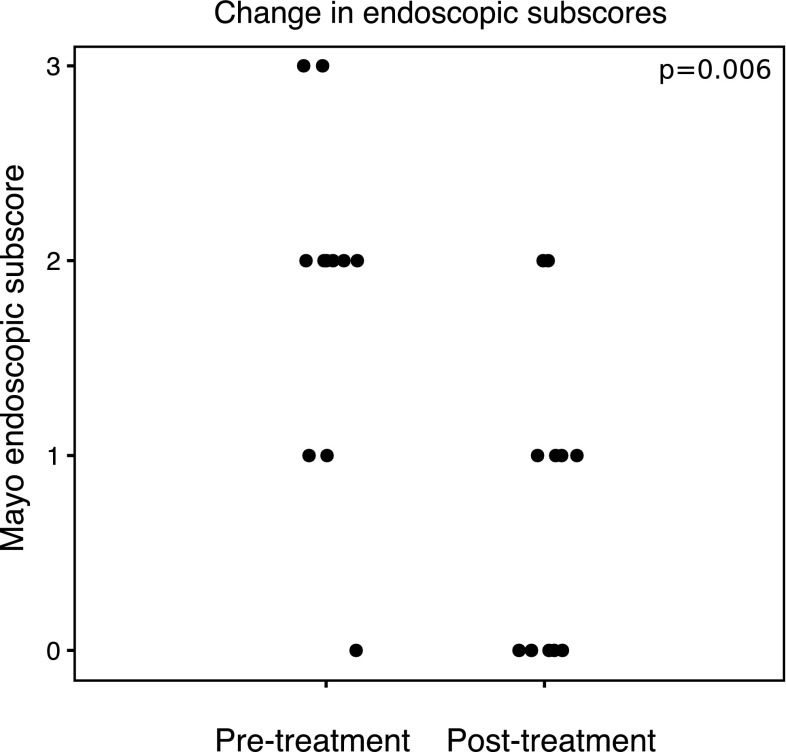
Pre-treatment and post-treatment Mayo endoscopic sub-scores following treatment with acetarsol suppositories

Clinical response was observed in 26 patients (68%). Of the 11 patients who had endoscopic assessment before and after treatment (Fig. 1), nine showed endoscopic improvement and four were in complete remission (Mayo endoscopic sub-score = 0, Wilcoxon signed-rank sum test *p* = 0.006). The only clinical parameter that correlated with clinical response was the number of previously failed immunomodulators (*p* = 0.01, mean 1.35 in responders and 2.46 in non-responders). Otherwise, there were no significant associations with response to treatment with respect to gender, IBD subtype, disease extent limited to the rectum, the presence of perianal fistula, previous failed medications use or concurrent medications (including corticosteroid, 5-ASAs, thiopurine and anti-TNFα drugs) after correction for multiple testing. Initial serum C-reactive protein (CRP) levels were not higher in responders compared with non-responders (mean CRP 12.5 mg/L vs. 10.5, respectively, *p* = 0.85).

Six patients (16%) with extensive disease underwent colectomy during the follow-up period, including two patients who had initially responded to acetarsol. Six patients required escalation of therapy: two went on to receive anti-TNFα therapy, two were enrolled in a clinical trial, one patient was commenced on tacrolimus enemas, and one responded to subsequent treatment with methotrexate. No patients in our cohort developed renal, cardiac or neurological disease during the follow-up period nor were any malignancies diagnosed.

One patient developed a moderate macular skin rash 1 week after commencing acetarsol suppositories. Treatment was stopped immediately and the rash resolved within 4 weeks. Skin biopsy confirmed superficial perivascular dermatitis, consistent with a drug rash.

## Discussion

In this paper, we describe the experience of a single tertiary referral center in the United Kingdom, where acetarsol suppositories have been used for the treatment of refractory symptoms of proctitis since 2004. Arsenic, a metalloid element that exists in both organic and inorganic forms, has been used in medicine and other industries for over 2400 years [10]. Acetarsol is formulated from organic arsenic, meaning that the arsenic atom is attached to hydrocarbon complexes. Organic arsenic is far less toxic than the inorganic form [11] and is commonly found in seafood [12]. Following consumption of lobster, for example, high levels of organic arsenic are measurable in urine [13]. In vitro testing has demonstrated organic arsenic compounds to be 50–600 times less toxic than inorganic arsenic compounds [14]. Exposure to inorganic arsenic, however, can lead to severe gastrointestinal, neurological and renal damage, and even death through cardiac arrhythmia [15]. Additionally, chronic exposure to inorganic arsenic, for example through contaminated groundwater or crops, is associated with skin lesions and an increased risk of certain malignancies (skin, bladder, and lung).

The mode of action of acetarsol is unclear. Conversely, more is known about inorganic arsenic, especially its potential anti-inflammatory and carcinogenic effects. miRNAs may play a role in the anti-inflammatory effects of arsenic and may have been implied in its carcinogenic potential [16–18], with reports linking it to urothelial and skin cancers among others. Inorganic arsenic is a potent inducer of MAP kinase signal transduction pathways. The differential activation of MAP kinase pathways may contribute to cell growth regulation and cell death in response to diverse doses of arsenic with high inorganic concentration leading to apoptosis and low concentrations resulting in enhanced cell proliferation and carcinogenesis [17]. Given that such significant toxicities have not been associated with organic arsenic, it is probable that its biological properties differ to those of inorganic arsenic.

In our clinical experience, patients respond quite rapidly to acetarsol suppositories, often within a matter of a few days. The response is sometimes temporary, and patients may require further courses of treatment. Even after relapse, patients usually responded to a second course. Of our cohort, six patients (15%) had multiple courses of acetarsol—up to four courses in total. All of these patients achieved a clinical response with the subsequent courses. It has been our practice to observe a 4-week “washout” period prior to a second course to avoid systemic arsenic exposure. However, we do not routinely measure serum arsenic levels. In their 1989 paper, Forbes et al. did measure serum and urine arsenic levels and demonstrated serum concentration approaching the toxic range (> 200 µg/L) during the first week of treatment in most patients. Serum levels decreased rapidly despite ongoing treatment. This probably reflects a reduction in systemic absorption of acetarsol as rectal inflammation decreases. Serum arsenic levels returned to baseline levels in all patients 4 weeks after treatment cessation. Our study was not designed to assess the safety of acetarsol. However, no patients in our cohort developed conditions consistent with arsenic toxicity in the follow-up period (mean 6.5 years). Nevertheless, as this was a retrospective analysis of medical records at a single institution, conditions diagnosed and treated elsewhere may have not been recorded. Acetarsol suppositories were well tolerated by our cohort, with only one patient experiencing an adverse reaction. This patient developed a moderate skin rash that settled promptly following drug withdrawal.

In 1965, Connell et al. published a double-blind study of 44 patients with proctitis [1]. The patients were randomized to receive two 5 mg prednisolone suppositories or two 250 mg acetarsol suppositories, nightly for 3 weeks. Twenty patients in each group were followed up and underwent sigmoidoscopy. Endoscopic improvement was seen in 19 patients in the acetarsol group, versus 17 in the prednisolone group. Symptomatic improvement was noted in 18 patients in the acetarsol group versus 15 of those treated with prednisolone. One patient in the acetarsol group experienced jaundice 2 weeks into treatment, which settled upon drug withdrawal. It should be noted that these patients were not a group refractory to other treatments and therefore represent a different cohort to our patients.

In our cohort, endoscopic evaluation was undertaken before and after treatment in 11 patients (Fig. 1). All four patients who were in endoscopic remission after the course of acetarsol suppositories (which we defined as a Mayo endoscopic sub-score of zero) had also achieved a clinical response. A further four patients had a post-treatment Mayo endoscopic sub-score of one, indicating only mild endoscopic disease. Similarly, all of these patients had achieved a clinical response.

Arsenic has similar chemical properties to bismuth, which is also a heavy metal used in the treatment of proctitis [19]. In a randomized double-blinded study of 63 patients with distal colitis, participants received either nightly enemas comprising of 5-ASA (2 g in 100 mL) or bismuth citrate carbomer (equivalent to 450 mg bismuth citrate) for 4 weeks. Clinical remission was observed in 18 of 32 patients in the 5-ASA group and 12 of 31 patients in the bismuth group (*p* = 0.16).

This study represents the largest cohort of patients treated with acetarsol suppositories reported to date and the first publication on this topic in over 25 years. We recognize the significant limitations of a retrospective analysis such as this. Formal Mayo scores, including sub-scores for rectal bleeding, stool frequency, and physician’s global assessment, were not possible due to the nature of the medical records. Thus, subjective definitions of clinical response and remission were used. However, we were able to objectively assign endoscopic Mayo sub-scores to a proportion of our patients. Not all patients underwent endoscopic evaluation in a timely manner following treatment; in fact, only 11 of 38 patients had a follow-up endoscopy at all, and this was performed at the discretion of the treating physician. The median time from the end of the treatment course until endoscopic evaluation was 5 months, raising doubt about the significance of the endoscopic findings. Patients who did undergo repeat endoscopy did so at the time of routine clinic visit, rather than in a protocolled manner to investigate the efficacy of acetarsol. Our cohort was typical of those with refractory proctitis. They had been affected by IBD for median 6.9 years (SD ± 5.0) and had tried a median 5.5 ± 1.2 different medications prior to the course of acetarsol. One quarter of our cohort had previously been treated with anti-TNFα therapy, and 82% had received prior thiopurine therapy.

In conclusion, acetarsol was effective in two out of every three patients with refractory proctitis. Our cohort had previously failed a broad range of topical and systemic treatments. The clinical efficacy reflected the significant endoscopic improvements. Adverse effects of acetarsol were rare. Further evidence, in the form of a prospective clinical trial, is required before recommending wider use of acetarsol for the treatment of refractory proctitis.
